# Structural Plasticity of Eph Receptor A4 Facilitates Cross-Class Ephrin Signaling

**DOI:** 10.1016/j.str.2009.07.018

**Published:** 2009-10-14

**Authors:** Thomas A. Bowden, A. Radu Aricescu, Joanne E. Nettleship, Christian Siebold, Nahid Rahman-Huq, Raymond J. Owens, David I. Stuart, E. Yvonne Jones

**Affiliations:** 1 Division of Structural Biology, University of Oxford, Henry Wellcome Building of Genomic Medicine, Roosevelt Drive, Oxford OX3 7BN, UK; 2 Oxford Protein Production Facility, University of Oxford, Henry Wellcome Building of Genomic Medicine, Roosevelt Drive, Oxford OX3 7BN, UK

**Keywords:** SIGNALING, CELLBIO

## Abstract

The EphA4 tyrosine kinase cell surface receptor regulates an array of physiological processes and is the only currently known class A Eph receptor that binds both A and B class ephrins with high affinity. We have solved the crystal structure of the EphA4 ligand binding domain alone and in complex with (1) ephrinB2 and (2) ephrinA2. This set of structures shows that EphA4 has significant conformational plasticity in its ligand binding face. In vitro binding data demonstrate that it has a higher affinity for class A than class B ligands. Structural analyses, drawing on previously reported Eph receptor structures, show that EphA4 in isolation and in complex with ephrinA2 resembles other class A Eph receptors but on binding ephrinB2 assumes structural hallmarks of the class B Eph receptors. This interactive plasticity reveals EphA4 as a structural chameleon, able to adopt both A and B class Eph receptor conformations, and thus provides a molecular basis for EphA-type cross-class reactivity.

## Introduction

Eph (Erythropoietin-producing hepatoma) tyrosine kinase cell surface receptors comprise the largest group of receptor tyrosine kinases (Hirai et al., 1987). These receptors with their membrane-bound ligands, the ephrins (Eph receptor interacting proteins), contribute to fundamental developmental processes such as boundary formation and cell migration in multicellular organisms ranging from sponges to vertebrates (Drescher, 2002; Kullander and Klein, 2002). The signals resulting from Eph-ephrin complex formation can be bidirectional (forward: into the Eph-presenting cell, reverse: into the ephrin-presenting cell) (Cowan and Henkemeyer, 2002; Lim et al., 2008; Lu et al., 2001).

There are fourteen Eph receptors and eight ephrin ligands in the human genome, each family divided into two classes (A and B) (Eph Nomenclature Committee, 1997). The extracellular portions of ephrinA ligands are attached to the cell by a glycosylphosphatidylinositol anchor, while ephrinB ligands contain a transmembrane helix and a short intracellular region. Eph receptors are type 1 membrane proteins with an N-terminal extracellular region consisting of a ligand binding domain (LBD), a cysteine rich region, and two fibronectin type III domains. The extracellular region is followed by a single transmembrane span connecting to a cytoplasmic region comprising a protein tyrosine kinase domain that mediates autophosphorylation and phosphorylation of other proteins (Pasquale, 2005), a sterile α motif (SAM), and a PDZ-binding motif.

Generally, it has been found that high affinity binding (nM to low μM K_D_) of Eph receptors to ephrin ligands is restricted within classes (i.e., class A ligands bind class A receptors) (Gale et al., 1996). However, this rule only holds to an extent. EphA4, for example, has been found to bind both to A and B class ligands and EphB2 has been found to bind to ephrinA5 (Gale et al., 1996; Himanen et al., 2004).

EphA4 is associated with an extensive range of biological activity; for example, it is critical for the development of the nervous system (Canty et al., 2006; Coulthard et al., 2002; Dottori et al., 1998; Fu et al., 2007), is also highly expressed during glioma cell proliferation (Fukai et al., 2008), and has been linked with melanoma tumor suppression (Easty and Bennett, 2000). The potential to target Eph receptors for cancer therapeutics is of growing interest. Structural studies of the LBD of EphB2 and EphA4, for example, have shown that these receptors may be useful targets for the design of small molecule cancer therapeutics (Chrencik et al., 2006b; Noberini et al., 2008; Qin et al., 2008).

Knowledge of the molecular determinants that define Eph receptor cross-reactivity and specificity is a powerful prerequisite for the rational development of Eph receptor- and ephrin-based disease treatments. In this study, we sought to elucidate the mechanism by which the LBD of the EphA4 cell surface receptor binds to both A and B class ephrin ligands at the molecular level. We present binding affinity data of EphA4 with both A and B class ephrins and report the structure of the LBD of EphA4 alone and in complex with the receptor binding domain (RBD) of ephrinA2 and ephrinB2. From our analysis of these structures, we suggest that the LBD of EphA4 is structurally plastic and, as a consequence, has a bimorphic A and B class Eph receptor nature. These data provide a molecular basis for how differing patterns in Eph-ephrin binding define properties that influence class-dependent Eph receptor specificity and promiscuity.

## Results

### Crystal Structure of EphA4 Ligand Binding Domain

**Figure 1:**
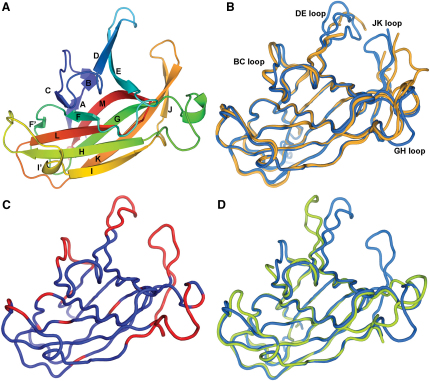
The Structure of the EphA4 LBD (A) Cartoon representation of the apo LBD of EphA4 colored as a rainbow with the N terminus in blue and the C terminus in red and with secondary structure labeled according to standard nomenclature. (B–D) Cα traces. (B) Superposition of all EphA4 molecules from the asymmetric unit of reported unbound crystal structures. Molecules in the asymmetric unit of the EphA4 structure we report here are colored blue, and molecules in the asymmetric unit of PDB code 3CKH are colored orange. (C) Representative EphA4 molecule colored according to regions of flexibility as defined by ESSET (Schneider, 2002). Regions colored blue are conformationally invariant and regions colored red are flexible. (D) Superposition of representative EphA4 (light blue) with crystal structure of unbound EphA2 (green; PDB code 3C8X).

The crystal structure of the uncomplexed (apo) EphA4 cell surface receptor LBD was solved and refined to 1.85 Å resolution (Table 1) with two molecules in the asymmetric unit, using EphB2 (Protein Data Bank [PDB] code 1NUK) as a model for molecular replacement. Similar to the previously reported apo-EphA2 (PDB code 3C8X) LBD (55% sequence identity in the LBD), EphA4 consists of 12 anti-parallel β strands (designated A–M) with two disulfide bonds and is arranged as a β sandwich (Figure 1A; see Figure S1 available online). We note that during the preparation of this manuscript an independently determined apo structure of EphA4 (PDB code 3CKH; 2.8 Å resolution) has been published and released in the PDB (Qin et al., 2008). Superposition of both molecules in the asymmetric unit of our high resolution structure with the two molecules in the asymmetric unit of PDB deposition 3CKH (overall 1.0 Å rms average deviation over 163 equivalent Cα atoms) reveals that there is a high degree of conformational plasticity in the regions of the BC, DE, GH, and JK loops (Figures 1B and 1C). The flexibility inherent in these regions is consistent with their relatively high average crystallographic main chain B factor values in comparison with the rest of the protein (for our high resolution structures an average of 62 Å^2^ in BC, DE, GH, and JK loops versus 31 Å^2^ over the entire molecule). Given that the DE and JK loops map to regions in EphA2 (Figure 1D) and EphB2 crystal structures (Himanen et al., 2001), which move upon binding to their respective ephrin ligands, their flexibility may be of direct functional relevance.

### EphA4 Displays a Broad Affinity Range for Ephrin Ligands

**Figure 2:**
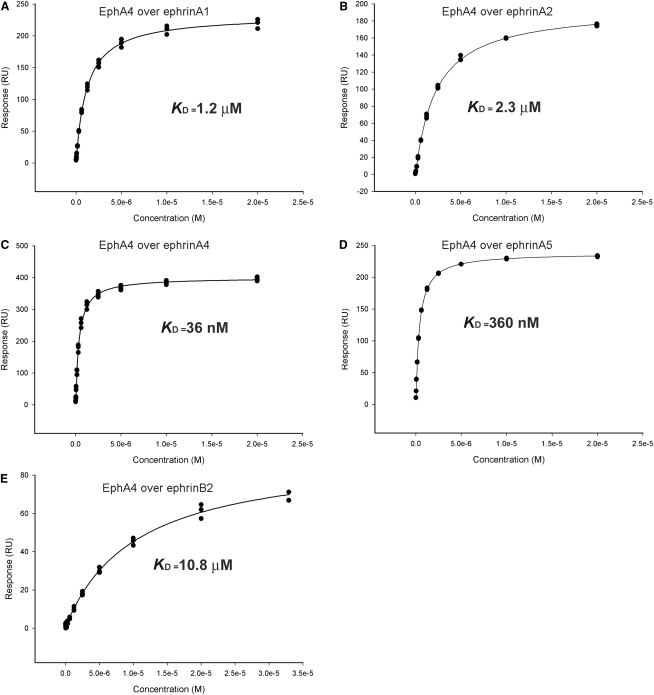
K_D_ Determination of EphA4 LBD with Ephrin RBD (A–E) Overlay association plots of surface plasmon resonance sensorgrams recording response of EphA4 (analyte) interaction with immobilized ephrinA1 (A), ephrinA2 (B), ephrinA4 (C), ephrinA5 (D), and ephrinB2 (E). Calculated affinity binding K_D_ values are shown.

To ascertain the strength of EphA4 binding to a panel of ephrins, affinity measurements of EphA4 with ephrinA1, ephrinA2, ephrinA4, ephrinA5, ephrinB1, and ephrinB2 were performed using surface plasmon resonance. Monomeric ephrin ligands were immobilized onto carboxymethyl (CM5) chip surfaces via a biotin-streptavidin linkage while monomeric EphA4 was used as an analyte with concentrations ranging from 20 nM to 30 μM. Consistent with previously reported fluorescence titration spectroscopy, isothermal calorimetry, and surface plasmon resonance studies that measured monomeric Eph-ephrin interactions, our data yielded Eph-ephrin binding constants in the nanomolar to low micromolar range (Figure 2) (Day et al., 2005; Himanen et al., 2004; Nikolov et al., 2005; Pabbisetty et al., 2007).

Our results show that the EphA4 receptor has a broad affinity range for different types of ephrin ligands with cross-class Eph receptor binding weaker (5–300 times) than EphA-ephrinA interactions. We find that EphA4 has greatest affinity for ephrinA4 (K_D_ = 36 nM), intermediate affinities to ephrinA1, ephrinA5, and ephrinA2 (K_D_ = 1.2 μM, K_D_ = 360 nM, and K_D_ = 2.3 μM, respectively), binds most weakly to ephrinB2 (K_D_ = 10.8 μM), and shows no detectable binding to ephrinB1.

### Crystal Structures of EphA4-ephrinB2 and EphA4-ephrinA2 Complexes

**Figure 3:**
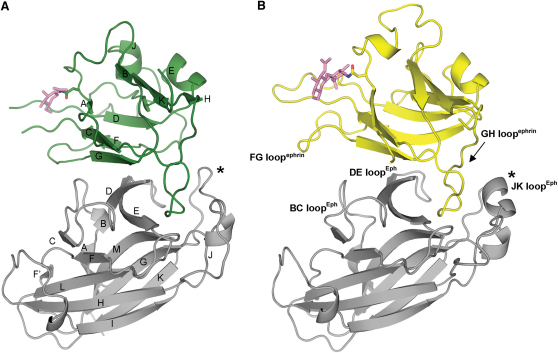
Crystal Structures of EphA4-ephrinB2 and EphA4-ephrinA2 (A and B) Cartoon representation of EphA4-ephrinB2 (A) and EphA4-ephrinA2 (B). Secondary structure elements are labeled according to standard nomenclature in A and the main interaction loops are labeled in B. The JK^Eph^ loops are marked with an asterisk and undergo major conformational changes dependent on the class of ephrin ligand bound. Acetyl-glucosamine moieties observed at N-linked glycosylation sites are shown as pink sticks.

In order to investigate the molecular mechanisms underlying EphA4 cross-reactivity, we sought to determine representative complexes with A and B class ephrins. The EphA4-ephrinB2 and EphA4-ephrinA2 complex structures were solved and refined to 2.4 and 2.3 Å resolution, respectively (Table 1). Our determination of these two structures increases the number of known Eph-ephrin complexes to six. The EphA4-ephrinB2 structure is the first cross-class EphA type complex. Both the overall fold and architecture of complex binding in these two structures remain consistent with previously reported Eph-ephrin complexes (Figure S2). EphA4 maintains the same β sandwich fold that is present in the unliganded structures, and the ephrin ligands retain a Greek key β barrel fold consisting of eight β strands (designated A–K) with hydrophobic residues in the GH^ephrin^ loop buried with almost no solvent accessibility between the DE^EphA4^ and JK^EphA4^ loops on EphA4 (Figure 3 and Figure S3).

To facilitate crystallization of the EphA4-ephrinA2 complex, a site-directed mutation, N174Q, was incorporated into the sequence of ephrinA2 to remove a glycosylation site not conserved between ephrins (Figure S4). As a result, only electron density corresponding to two reducing-terminal *N*-acetylglucosamine residues of an N-linked sugar at Asn42^ephrinA2^ was observed in the EphA4-ephrinA2 structure (Figure S5). In the EphA4-ephrinB2 structure, electron density corresponding to a single *N*-acetylglucosamine moiety was observed at Asn36^ephrinB2^ (conserved with Asn42^ephrinA2^) but not Asn139^ephrinB2^ (Figure S5).

The first structure of an Eph receptor-ephrin complex (EphB2-ephrinB2) (Himanen et al., 2001) revealed a heterotetrameric arrangement of molecules suggestive of a clustering mode involved in signaling. However, two subsequent complexes (EphB2-ephrinA5 and EphB2-ephrinB2) (Chrencik et al., 2006a; Himanen et al., 2004) have not shown such higher order arrangements and we do not observe any evidence of heterotetramerization in our structures of EphA4-ephrinB2 and EphA4-ephrinA2.

The overall total buried surface areas for the receptor-ligand interface in EphA4-ephrinB2 and EphA4-ephrinA2 are 2590 and 1930 Å^2^, respectively (Krissinel and Henrick, 2007). These values are comparable to those observed in the EphA2-ephrinA1 (PDB code 3CZU), EphB2-ephrinB2 (PDB code 1KGY), and EphB4-ephrinB2 (PDB code 2HLE) structures (buried surface areas of 2260, 2380, and 2120 Å^2^, respectively), but are in contrast to EphB2-ephrinA5 (PDB code 1SHW; buried surface area of 1220 Å^2^), the only other cross-class complex crystallized. Our EphA4-ephrinB2, EphA4-ephrinA2, and the recently deposited EphA2-ephrinA1 structures also have relatively high surface complementarity scores (Lawrence and Colman, 1993) (0.71, 0.76, and 0.73, respectively), which distinguishes them from the previously reported EphB2-ephrinB2, EphB4-ephrinB2, and EphB2-ephrinA5 structures (surface complementarity scores of 0.67, 0.64, and 0.66, respectively).

### Structural Rigidity of the Ephrin Ligand

**Figure 4:**
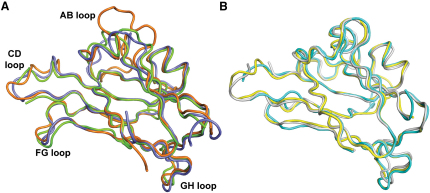
Structural Alignment of Ephrin Ligands in Their Bound States Ephrin A class ligands (A) and ephrinB2 ligands (B) are shown in cartoon representation. EphrinA1 from EphA2-ephrinA1 (PDB code 3CZU) is colored green, ephrinA2 from EphA4-ephrinA2 is colored orange, and ephrinA5 from EphB2-ephrinA5 (PDB code 1SHW) is colored blue. EphrinB2 from EphA4-ephrinB2 is colored cyan, ephrinB2 from EphB2-ephrinB2 (PDB code 1KGY) is colored yellow, and ephrinB2 from EphB4-ephrinB2 (PDB code 2HLE) is colored gray.

Comparison of ephrin ligands from multiple Eph-ephrin structures reveals that both A and B class ephrins are structurally invariant and independent of their Eph receptor pair (Figure 4). Upon detailed inspection, it is apparent, however, that the AB loop region is variable in A class ephrins. This is due to a six amino acid insertion in ephrinA2 that is not present in other ephrin ligands. As this region is remote from the Eph receptor binding face, it is unlikely to influence Eph binding.

Due to the high structural rigidity of ephrin ligand structures, we suggest that the overall binding conformation of an Eph receptor is molded by the identity of its cognate ligand. However, ephrinB2 can show structural plasticity in a different context; the conformation of the GH^ephrinB2^ loop is altered upon binding to the Nipah virus and Hendra virus attachment glycoproteins (Bowden et al., 2008a, 2008b).

### Plasticity of Eph Receptor A4 at the Ephrin Binding Interface

**Figure 5:**
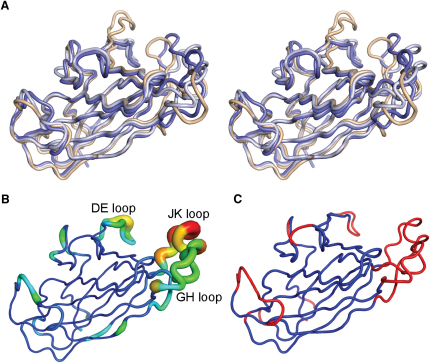
Conformational Changes of EphA4 upon Binding (A) Superposition of unbound EphA4 (orange), with its ephrinB2 (dark blue)- and ephrinA2 (gray)-bound states shown in stereo representation. (B) Rms displacement of equivalent residues between unbound, ephrinB2-bound, and ephrinA2-bound EphA4 mapped onto the Cα trace structure of EphA4 receptor (from EphA4-ephrinA2). The tube radius and color of the trace represent the rms displacement (ramped from blue to red). Regions with high deviations between structures are thick and colored red, while regions with low deviation are thin and colored blue. (C) Cα trace of EphA4 from EphA4-ephrinA2 colored according to regions of flexibility as defined by ESSET (Schneider, 2002). Regions colored blue are conformationally invariant and regions colored red are flexible.

**Figure 6:**
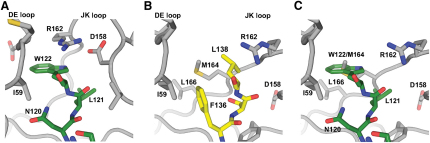
EphrinB2 Precludes the Formation of an α Helix in the JK^EphA4^ Loop upon Binding (A) Residues from the GH^ephrinB2^ loop (stick representation with nitrogen blue, oxygen red, and carbon green) bind between the DE^EphA4^ and JK^EphA4^ loops (stick representation with nitrogen blue, oxygen red, sulfur gold, and carbon gray). (B) Residues from the GH^ephrinA2^ loop (stick representation with nitrogen blue, oxygen red, and carbon yellow) bind between the DE^EphA4^ and JK^EphA4^ loops (gray van der Waals surface; stick representation with nitrogen blue, oxygen red, and carbon gray). (C) W125^ephrinB2^ from the GH^ephrinB2^ loop (green sticks, colored as in [A]) clashes with M164^EphA4^ when superposed onto EphA4 in its ephrinA2-bound state.

While ephrins generally exhibit a low degree of structural variance between different bound forms, structural analysis of EphA4 reveals distinct structural differences between unbound EphA4 and its ephrinA2 (1.7 Å rmsd over 166 equivalent Cα atoms)- and ephrinB2 (2.3 Å rmsd over 168 equivalent Cα atoms)-bound states (Figure 5A). Most notable are the structural changes that occur to the DE^EphA4^ and JK^EphA4^ loops (Figures 5B and 5C); regions which we identify above as having a high degree of flexibility in unbound EphA4. While the DE^EphA4^ loop moves appreciably between its unbound and bound states, its main chain conformation is maintained (0.5 Å rmsd over Cα atoms of residues Ser58^EphA4^-Arg68^EphA4^) between the ephrinA2- and ephrinB2-bound structures (Figure S6). This suggests that the DE^EphA4^ loop interacts in a conserved manner that is independent of the identity of the ephrin ligand. In contrast, the JK^EphA4^ loop is very different between its two bound states (2.8 Å rmsd over Cα atoms of residues Ala150^EphA4^-Leu166^EphA4^); forming an extended conformation when bound to ephrinB2 and an α helix (Gln156^EphA4^-Asp161^EphA4^) when bound to ephrinA2. The extended conformation of the JK^EphA4^ loop when bound to ephrinB2 appears to be stabilized by a hydrophobic stacking interaction between Arg162^EphA4^ and Trp122^ephrinB2^ (Figure 6A; Trp122 is equivalent to Trp125 in mouse). It is likely that this stacking interaction will be conserved in the interaction of EphA4 with ephrinB3 (Trp122 is conserved). These results also provide a rationale for why EphA4 does not bind to ephrinB1. Trp122^ephrinB2^ (equivalent to Met122^ephrinB1^) is not conserved and Tyr121^ephrinB1^ (equivalent to Leu121^ephrinB2^) is likely to clash with the JK^EphA4^ loop. The Arg162^EphA4^-Trp122^ephrinB2^ stacking interaction contrasts with that observed in the EphA4-ephrinA2 structure, where Arg162^EphA4^ is involved in hydrogen bonding interactions with the carbonyl oxygen of Leu138^ephrinA2^, while Met164^EphA4^and Leu166^EphA4^ participate in hydrophobic interactions with Leu138^ephrinA2^ and Phe136^ephrinA2^ (Figure 6B).

Although the JK^EphA4^ loop is α-helical in the EphA4-ephrinA2 structure, sequence-based secondary structure prediction programs (Bryson et al., 2005) predict the JK^EphA4^ loop region to be a coil or a turn. However, we note that this helix is not unprecedented and is analogous to that existing in the recently released EphA2-ephrinA1 structure (PDB code 3CZU; residues Ser153-Ala158^EphA2^). It is therefore likely that this helix is a structural motif specific to A class Eph receptor-ligand interactions and its formation is dependent on the nature of residues present in the GH^ephrin^ binding loop. This helix is not present in the EphA4-ephrinB2 structure due to the steric clash that would occur between Trp122^ephrinB2^ (rather than Leu138^ephrinA2^) and Met164^EphA4^ (Figure 6C). As a result, we suggest that the plasticity of the JK^EphA4^ loop region allows EphA4 to physically adapt to Trp122^ephrinB2^ and the resulting complementary stacking interactions with Arg162^EphA4^ are compensatory.

**Figure 7:**
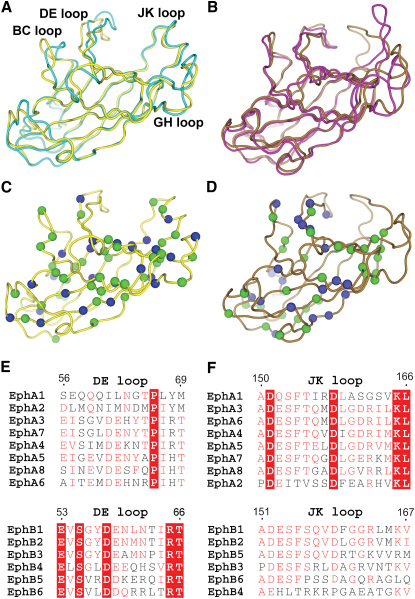
Structure and Sequence Conservation of EphA-ephrinA and EphB-ephrinB Complexes (A) Superposition of EphA4 (yellow) and EphA2 (cyan) from their ephrinA2- and ephrinA1-bound states, respectively. (B) Superposition of EphB2 (brown; PDB code 1KGY) and EphB4 (magenta; PDB code 2HLE) from their ephrinB2-bound states. (C and D) EphA4 from its ephrinA2-bound state (C; yellow) and EphB2 from its ephrinB2-bound state (D; brown; PDB code 1KGY) with sequence conservation mapped onto the structure as blue (residues completely conserved between EphA sequences) and green (residues at least 80% conserved across Eph sequences) spheres. (E) Sequence alignment of the DE^Eph^ loop numbered according to EphA4. (F) Sequence alignment of the JK^Eph^ loop numbered according to EphA4.

These observations prompted us to undertake a full comparison of EphA-ephrinA- and EphB-ephrinB-type complexes to detect any additional class-dependent trends (Figure 7). Superposition of EphA4 and EphA2 from their ephrinA2- and ephrinA1-bound structures, respectively, reveals that the JK^Eph^ α helix is well conserved both in structure and in amino acid sequence (Figures 7A, 7C, and 7F). This contrasts noticeably with the lack of sequence identity and varied extended coil conformations present in EphB2 and EphB4 when in complex with ephrinB2 (Figures 7B, 7D, and 7F). It is striking that the reverse observations can be made with the DE^Eph^ loop. EphB-ephrinB complexes demonstrate both a conserved structure and sequence (Figures 7B, 7D, and 7E), while EphA-ephrinA class complexes are structurally dissimilar and have relatively low sequence identity (Figures 7A, 7C, and 7E). On the basis of markedly different trends in sequence and structural conservation in these regions, we suggest that the DE^EphA4^ and JK^EphA4^ loops provide a mechanism by which A and B class Eph receptors, respectively, generate specificity and cross-class reactivity.

### The Architecture of Complex Binding is Dependent on the Ephrin Ligand

**Figure 8:**
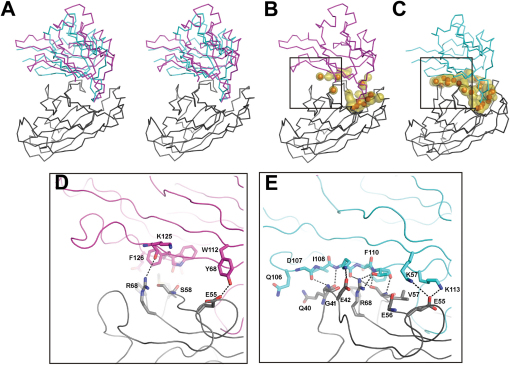
The Extended Ephrin Binding Site (A–E) Eph and ephrin Cα traces where EphA4 is colored gray, ephrinA2 is colored pink, and ephrinB2 is colored cyan. (A) Comparison of ephrinA2 (pink)- and ephrinB2 (cyan)-bound to EphA4 (gray) based on superposition of the EphA4 component (shown in stereo representation) reveals a 12° relative tilt between ephrinA- and ephrinB-bound complexes. (B and C) The presence or absence of an extended Eph-ephrin binding surface correlates with the degree of van der Waals and hydrogen bonding interactions. Midpoints between van der Waals interactions are shown as gold surfaces and intracomplex hydrogen bonds are shown as red spheres in the EphA4-ephrinA2 (B) and EphA4-ephrinB2 (C) complexes. Boxes in (B) and (C) refer to areas highlighted in (D) and (E). Colored as in (A). (D and E) Van der Waals and hydrogen bonding interactions taking place in the extended binding surface. Residues involved in protein-protein contacts are shown as sticks where carbon atoms are colored as in (A)–(C), nitrogen atoms are colored blue, oxygen red, and carbon yellow. Dashed lines correspond to intracomplex hydrogen bonds.

Superposition of our EphA4 complexes reveal that the relative orientation of ephrinA2 when bound to EphA4 differs by a tilt of 12° in comparison to the ligand in the EphA4-ephrinB2 structure (Figure 8A) and results in fewer intracomplex interactions beyond the DE^EphA4^ and JK^EphA4^ surface channel (Figures 8B and 8C). The observation that ephrinA-type ligands bind to their respective Eph receptor with a different tilt, which therefore results in a lack of an extended binding site, has been made by previous comparison of the EphB2-ephrinA5, EphB2-ephrinB2, and EphB4-ephrinB2 structures (Himanen et al., 2007) and holds true for the recently deposited EphA2-ephrinA1 structure (Figure S7). The presence or absence of an extended binding site correlates with the van der Waals and hydrogen bonding patterns (Figures 8D and 8E and Figure S7). While the EphA4-ephrinB2 interface is comprised of 15 hydrogen bonds and a total of 30 residues participating in van der Waals interactions, EphA4-ephrinA2 contains only 6 and 22, respectively. This ephrin class difference is also consistent with previously reported structures where EphB2-ephrinB2, EphB2-ephrinB4, EphA2-ephrinA1, and EphB2-ephrinA5 have 14, 12, 8, and 2 hydrogen bonds, respectively, which lie away from the hydrophobic surface channel formed by the DE^EphA4^ and JK^EphA4^ loops. Although these observations do not take into account differences in crystal packing, these patterns suggest a second characteristic dissimilarity between class A and B ephrin binding.

### Structural Phylogenetic Analysis of Multiple Eph Receptors and Ephrin Ligands

**Figure 9:**
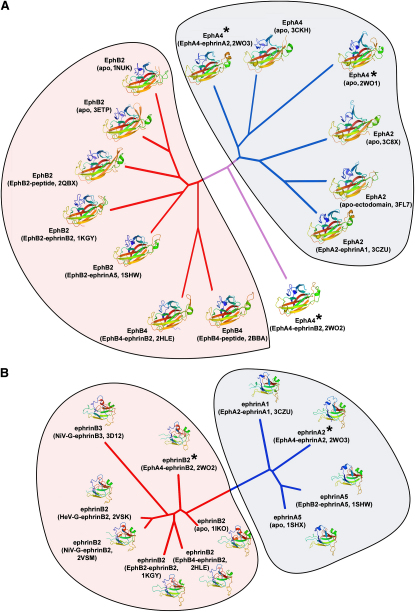
Structure-Based Phylogenetic Analysis of Eph Receptors and Ephrin Ligands (A and B) Unrooted trees of the Eph receptor LBD (A) and ephrin ligand RBD (B). The structures were superposed and a pairwise distance matrix was constructed as previously described (Graham et al., 2008). PDB codes for each representative structure are labeled beneath a cartoon representation ramped from blue (N terminus) to red (C terminus). Branches colored blue correspond to A class Eph and ephrin families, branches colored red correspond to B class Eph and ephrin families, and the branch colored lilac (A) corresponds to EphA4 from its ephrinB2-bound state, which does not belong to either family. Structures marked with an asterisk are those presented within this manuscript.

Given the recent availability of multiple high resolution Eph and ephrin crystal structures, we performed structure-based phylogenetic analyses on the LBD of the Eph receptor and RBD of the ephrin ligand (Figure 9). A structural-based phylogenetic tree was derived with SHP (Stuart et al., 1979) and plotted with PHYLIP (Felsenstein, 1989) using all currently available Eph receptors and ephrin ligands (from 14 and 11 PDB entries, respectively). Similar to a sequence-based phylogenetic analysis (Figure S8), Eph receptors (Figure 9A) and ephrin ligands (Figure 9B) are structurally divided into two different branches dependent on their A or B type classification. Conspicuously, in the Eph receptor tree (Figure 9A), EphA4 in its ephrinB2-bound state is the most structurally similar out of all A class Eph receptors to the B class Eph receptors. Other EphA receptor structures, including the apo and ephrinA2-bound EphA4 structures, on the other hand, are more distantly related to B class Eph receptors. It appears that EphA4 maintains an element of conformational duality and flexibility that allows it to act as a pseudo class B Eph receptor, but, in corroboration with our surface plasmon resonance binding data, at an energetic cost with reduced binding affinity. The link between structure and cross-class promiscuity, however, is more difficult to establish with EphB2, the other known cross-class, high-affinity binding receptor. In contrast to the relatively large structural distance between different forms of the EphA4 LBD, EphB2 is much less structurally variant and, as a result, these structures cluster together much more closely.

## Discussion

Here, we have presented equilibrium binding data and crystal structures of the apo, ephrinB2-bound, and ephrinA2-bound forms of the cross-class reactive EphA4 LBD. Our surface plasmon resonance data show that EphA4 interacts with ephrin ligands with a broad affinity range. Eph receptors and their ephrin ligands are known to be expressed in complementary gradients (Hafner et al., 2004), a characteristic that appears consistent with receptor binding spanning a wide range of ligand concentrations. We suggest that this wide affinity range reflects a mechanism of fine tuning the response to multiple ligands encountered and perhaps different physiological functions.

From our structural analysis, we do not detect any indication of higher order EphA-ephrin heterotetramers. The structural differences between the two available apo LBD structures of EphA4 and the divergence in conformational changes that occur upon ephrinB2 and ephrinA2 binding are consistent with the EphA4 receptor maintaining a high degree of structural plasticity in its DE^EphA4^ and JK^EphA4^ ephrin binding loops. These changes include the introduction of unpredicted α-helical secondary structure in the JK^EphA4^ loop of the EphA4-ephrinA2 complex. Based on the high degree of sequence conservation in the JK loop across all A class Eph receptors, it is likely that this α helix motif is common to all EphA-ephrinA complexes. In contrast, in EphB-ephrinB complexes, the DE loop is more conserved in both sequence and structure. Therefore, we suggest that these two structural features are class-specific characteristics that distinguish between EphA-ephrinA and EphB-ephrinB modes of interaction.

Based on their structures of complexed and uncomplexed EphA2, Himanen et al. (2009) have very recently proposed that A class Eph receptor-ephrin interactions can be described by a “lock-and-key” mechanism. However, inspection of the EphA2 unbound structures deposited by the Structural Genomics Consortium (Toronto) indicates that for these crystal structures the primary EphA2 ligand binding loops (JK and DE loops) are partially disordered or are in conformational states quite different to their bound forms. These properties are similar to those observed in our EphA4 structures and the previously reported unbound EphA4 structure (Qin et al., 2008), results that suggest that the ligand binding surface of the EphA receptor is molded by the ephrin ligand. In light of these observations, although we cannot discount a lock-and-key mechanism until a greater range of A class Eph receptor structures are available, the evidence that A class Eph receptors are more likely than B class Eph receptors to be structurally rigid between their bound and unbound states is still inconclusive.

The bidirectional downstream signaling processes that result from Eph receptor-ephrin ligand binding regulate a range of biological processes from axon guidance to vascular development. The specific biology is dependent on the identity of the Eph receptor-ephrin ligand pair (Pasquale, 2005). By decoding the molecular basis for specificity and promiscuity of binding, we can aim to understand the hierarchy of Eph-ephrin interactions.

## Experimental Procedures

### Cloning and Expression

Human ephrinA2 (residues 33–177; GenBank accession number NP_001396) and human ephrinB2 (residues 27–167; GenBank accession number NP_004084) were cloned into the pHLSec vector (Aricescu et al., 2006b) and human EphA4 (residues 30–202; GenBank accession number NP_004429) was cloned into the pOPING vector (Berrow et al., 2007). Both vectors encode an optimal signal peptide for HEK293T cell-based expression and a C-terminal Lys-His6 tag. Proteins were expressed transiently in HEK293T cells in the presence of the class I α-mannosidase inhibitor kifunensine (1 mg L^−1^ culture). Transfections were performed with polyethyeleneimine either with a single DNA construct or as cotransfections for complex formation.

### Purification

EphA4 alone and in complex with ephrinA2 and ephrinB2 was purified by immobilized metal affinity chromatography using nickel-charged chelating Sepharose beads (GE Healthcare). EphA4-ephrinB2 complex was treated with endoglycosidase F1 (75 μg mg^−1^ protein, 12 hr, 21°C) to cleave glycosidic bonds of N-linked sugars within the di-*N*-acetylchitebiose core. Following deglycosylation, protein complexes were purified by size exclusion chromatography using a Superdex 75 10/30 column (Amersham) in 150 mM NaCl and 10 mM Tris (pH 7.5–8.0) buffer. The typical yield of purified protein (or protein complex) was approximately 2.5 mg complex L^−1^ culture, and cotransfection of EphA4 with ephrinB2 resulted in an approximately 50% increase in overall yield (4.0 mg complex L^−1^).

### Mutagenesis of N-linked Glycosylation Sites on EphrinA2

Mutagenesis of the potential N-linked glycosylation site N174Q was achieved by incorporation of the desired mutation in the C-terminal primer used for PCR cloning (Pyrobest Polymerase, Takara). Following PCR, the product was cloned into the pHLsec vector.

### Crystallization, Data Collection, Structure Determination, and Refinement

Protein crystals were grown using the sitting drop vapor diffusion method using 100 nL protein plus 100 nL precipitant according to previously described methods (Walter et al., 2005) (Table S1). Crystals were flash frozen by immersion of the crystal into a cryoprotectant containing glycerol, PFO-X125/03 (Lancaster Synthesis), or PEG (polyethylene glycol) 400 followed by rapid transfer to a gaseous nitrogen stream at 100 K. Data were collected at the European Synchrotron Radiation Facility (ESRF) beamline BM-14 and Diamond beamline I03. Images were integrated and scaled using the programs DENZO and SCALEPACK (Otwinowski and Minor, 1997). Crystallographic statistics are presented in Table 1. For all structures, 5% of reflections were randomly set aside to calculate the R_free_. Structures were solved by molecular replacement using the program Phaser (Storoni et al., 2004). The structure of EphA4 was solved using EphB2 (PDB code 1NUK) as a molecular replacement model and the structures of EphA4 in complex with ephrinB2 and ephrinA2 were solved using the EphB2-ephrinB2 structure (PDB code 1KGY). Initial model building for the high resolution EphA4 structure was performed with the program ARP-wARP (Perrakis et al., 2001). Structure refinement included iterative model building using Refmac 5 (Murshudov et al., 1997). Refinement cycles included rigid body refinements and restrained refinement with TLS. The molecular graphics program COOT was used for manual rebuilding (Emsley and Cowtan, 2004). The program MolProbity was used to validate models (Davis et al., 2007).

### Surface Plasmon Resonance Binding of EphA4 to Ephrin Ligands

The RBDs of human ephrinA1 (residues 16–158; GenBank accession number AAH32698), ephrinA2 (residues 33–174; GenBank accession number NP_001396), ephrinA4 (residues 23–162; GenBank accession number AAI07484), ephrinA5 (residues 27–166; GenBank accession number NP_001953), ephrinB1 (residues 26–170; GenBank accession number NP_004420.1), and ephrinB2 (residues 25–168; GenBank accession number NP_004429) were subcloned into the pHLsec-avitag-3 vector (Aricescu et al., 2006b). Ephrin ligands were immobilized on chelating Sepharose Fast Flow Ni^2+^-agarose beads (GE Healthcare) and biotinylated as described previously (Aricescu et al., 2006a). All ephrin ligands used for these binding studies had wild-type glycosylation. Affinity measurements between ephrin ligands and EphA4 receptor were performed using a BIAcore T100 (Biacore). Purified, biotinylated ephrin ligands were immobilized on a CM5 BIAcore sensor chip (Biacore) coated with streptavidin (Sigma). A surface with only streptavidin present on the chip was reserved to subtract background responses due to differences in refractive indices of running and sample buffers. For affinity measurements of EphA4 analyte binding to immobilized ephrin ligands, samples were injected at 20 μl min^−1^ (25°C) in HBS-EP buffer (10 mM HEPES [pH 7.4], 150 mM NaCl, 0.005% surfactant P20, and 3 mM EDTA). To calculate binding affinity, several cycles of association and dissociation at different concentrations of analyte were required. Analyte concentrations ranged from 20 nM to 30 μM. K_D_ values were calculated using Biacore Evaluation Software Version 1.1.

### Superpositions and Illustrations

All molecular superpositions were calculated using SHP (Stuart et al., 1979). All molecular representations were produced with Pymol (http://pymol.sourceforge.net). Sequence alignments were performed with MultAlin (Corpet, 1988) and formatted with ESPript (Gouet et al., 1999). Figures were prepared using Adobe Illustrator, Adobe Photoshop, CorelDraw, and Microsoft Publisher.

## Figures and Tables

**Table 1:** Data Collection and Refinement Statistics

Data Collection	EphA4	EphA4-ephrinA2	EphA4-ephrinB2
Beamline	ESRF BM14	Diamond I03	ESRF BM14
Resolution (Å)	25.0–1.85 (1.92–1.85)	50.0–2.35 (2.43–2.35)	30.0–2.45 (2.54–2.45)
Spacegroup	P2_1_	P6_4_	P6_1_
Cell dimensions (Å) and angles (°)	*a* = 48.7, *b* = 73.2,	*a* = 115.4, *b* = 115.4,	*a* = 107.4, *b* = 107.4,
	*c* = 54.4,	*c* = 59.3,	*c* = 47.6,
	β = 98.3	γ = 120	γ = 120
Wavelength (Å)	0.977	1.810	0.977
Unique reflections	31875 (3127)	18961 (1872)	11645 (1084)
Completeness (%)	98.2 (97.4)	99.9 (99.4)	99.3 (94.2)
R_merge_ (%)^a^	0.069 (0.344)	0.091 (0.749)	0.054 (0.531)
I/σI	19.5 (4.0)	30.0 (1.8)	43.2 (2.6)
Average redundancy	3.7 (3.7)	16.0 (8.1)	11.7 (6.6)
Refinement			
Resolution range (Å)	30.0–1.85 (1.90–1.85)	50.0–2.35 (2.41–2.35)	28.0–2.45 (2.51–2.45)
Number of reflections	30277	17987	11062
R_work_ (%)^b^	18.7	19.8	21.6
R_free_ (%)^c^	23.4	24.5	26.0
Rmsd bonds (Å)	0.010	0.011	0.006
Rmsd angles (°)	1.2	1.3	1.0
Rmsd main chain bond B (Å^2^)	0.8	0.8	0.3
Rmsd side chain bond B (Å^2^)	1.8	1.6	0.7
Rmsd between NCS related Cα atoms (Å)	0.5	NA	NA
Number of atoms per asymmetric unit (protein/water/sugar/ligand^d^)	2936/296/NA/20	2576/136/28/NA	2533 /49/14/NA
Average B factors (protein/water/sugar/ligand^d^) (Å^2^)	31.4/27.0/NA/40.2	43.7 /25.1/47.9/NA	49.9/22.2/57.2/NA
Model quality			
Ramachandran (%) (favored, additionally allowed, outlier)	94.2, 5.5, 0.3	97.5, 2.5, 0	96.4, 3.6, 0

Numbers in parentheses refer to the relevant outer resolution shell. Rmsd, root mean square deviation from ideal geometry.

a R_merge_ = Σ_hkl_ Σ_i_|*I(hkl;i)* − < *I(hkl)* > |/Σ_hkl_ Σ_i_*I(hkl;i)*, where *I(hkl;i)* is the intensity of an individual measurement and < *I(hkl)* > is the average intensity from multiple observations.

b R_factor_ = Σ_hkl_‖*F*_obs_| − *k*|*F*_calc_‖/Σ_hkl_ |*F*_obs_|.

c R_free_ is calculated as for R_work_, but using only 5% of the data that was sequestered prior to refinement.

d Isopropan-1-ol was observed in the crystal structure and was derived from the screening condition.

## Accession Numbers

Atomic coordinates and structural factors have been deposited in the Protein Data Bank with accession codes 2WO1 (EphA4), 2WO2 (EphA4-ephrinB2), and 2WO3 (EphA4-ephrinA2).

## Supplemental Data

Document S1. Eight Figures, One Table, and Supplemental References
