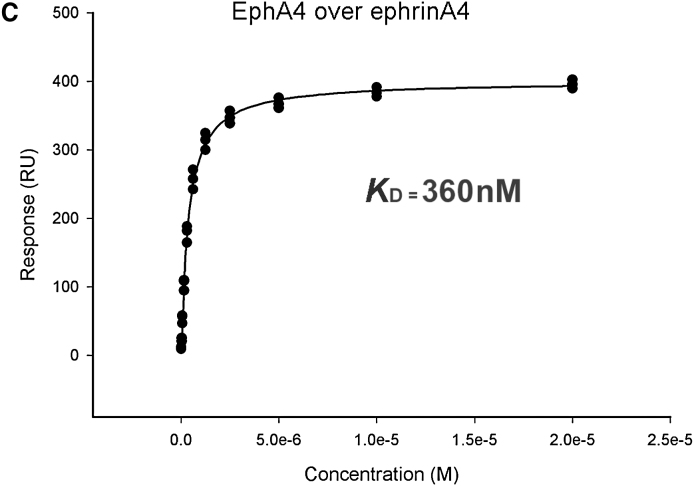# Structural Plasticity of Eph-Receptor A4 Facilitates Cross-Class Ephrin Signaling

**DOI:** 10.1016/j.str.2009.11.004

**Published:** 2009-12-09

**Authors:** Thomas A. Bowden, A. Radu Aricescu, Joanne E. Nettleship, Christian Siebold, Nahid Rahman-Huq, Raymond J. Owens, David I. Stuart, E. Yvonne Jones

(Structure *17*, 1386–1397; October 2009)

Due to an author error, an affinity plot in Figure 2 was inadvertently mislabeled. The stated K_D_ for panel (C) should be “360 nM”; the corrected Figure 2C is printed below. As a result of this correction, the second paragraph of page 1388 should read as follows:

Our results show that the EphA4 receptor has a broad affinity range for different types of ephrin ligands with cross-class Eph receptor binding weaker (5–30 times) than EphA-ephrinA interactions. We find that EphA4 has greatest affinity for ephrinA4 (K_D_ = 360 nM ± 20 nM) and ephrinA5 (K_D_ = 360 nM ± 10 nM), intermediate affinities to ephrinA1 and ephrinA2 (K_D_ = 1.2 μM ± 0.1 μM and K_D_ = 2.3 μM ± 0.1 μM, respectively), binds most weakly to ephrinB2 (K_D_ = 10.8 μM ± 2.1 μM), and shows no detectable binding to ephrinB1.

We apologize for this error, which, however, has no impact on any of the conclusions drawn in this article.

**Figure:**