# Supplementary material for: Structural Plasticity of Eph Receptor A4 Facilitates Cross-Class Ephrin Signaling
Source: Structure. 2009 Oct 14;17(10):1386–97. doi: 10.1016/j.str.2009.07.018 (PMC2832735; doi:10.1016/j.str.2009.07.018)
Supplement: Document S1. Eight Figures, One Table, and Supplemental References [file mmc1.pdf]

Supplemental Data

Structural Plasticity of Eph Receptor A4 Facilitates Cross-Class Ephrin Signaling

Thomas A. Bowden, A. Radu Aricescu, Joanne E. Nettleship, Christian Siebold, Nahid Rahman-Huq, Raymond J. Owens, David I. Stuart, and E. Yvonne Jones

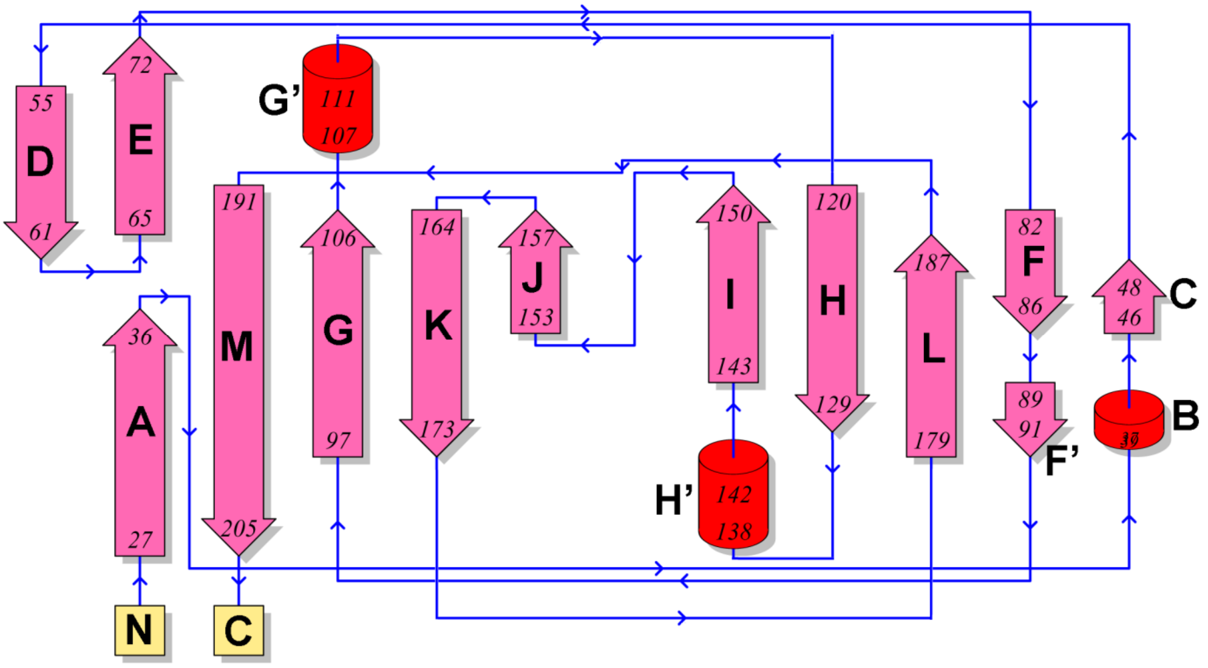

Figure S1. Topology of EphA4
Topology diagrams were generated using PDBsum (Laskowski, 2009) labelled according to standard Eph nomenclature with secondary structure elements shown as an arrow (β-strand) or helix (α helix).

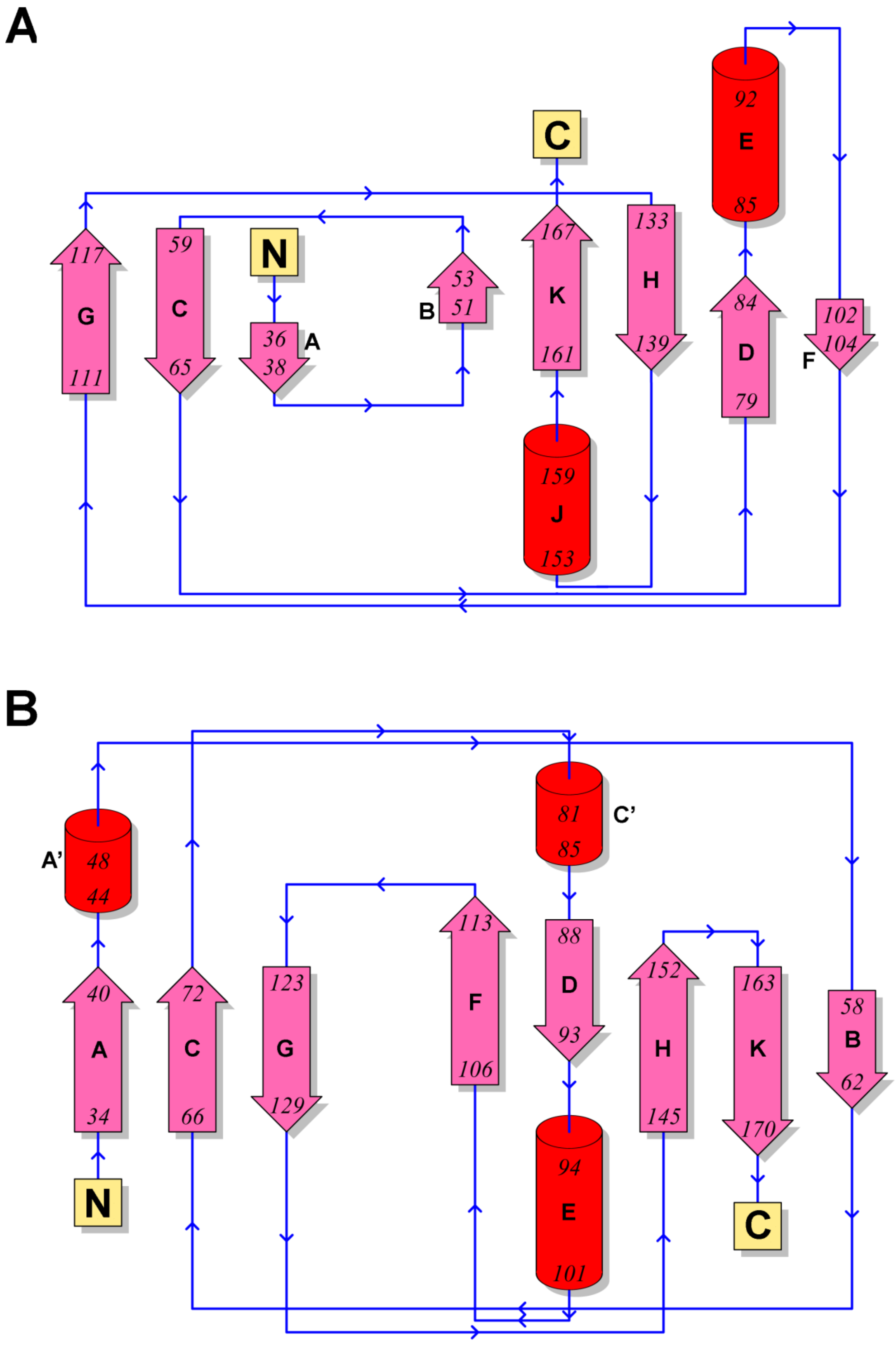

Figure S2. Topology of ephrinB2 and ephrinA2 when Bound to EphA4
(A and B) EphrinB2 (A) and ephrinA2 (B) are shown and labelled according to standard ephrin nomenclature with secondary structure elements depicted and generated as described in Figure S1.

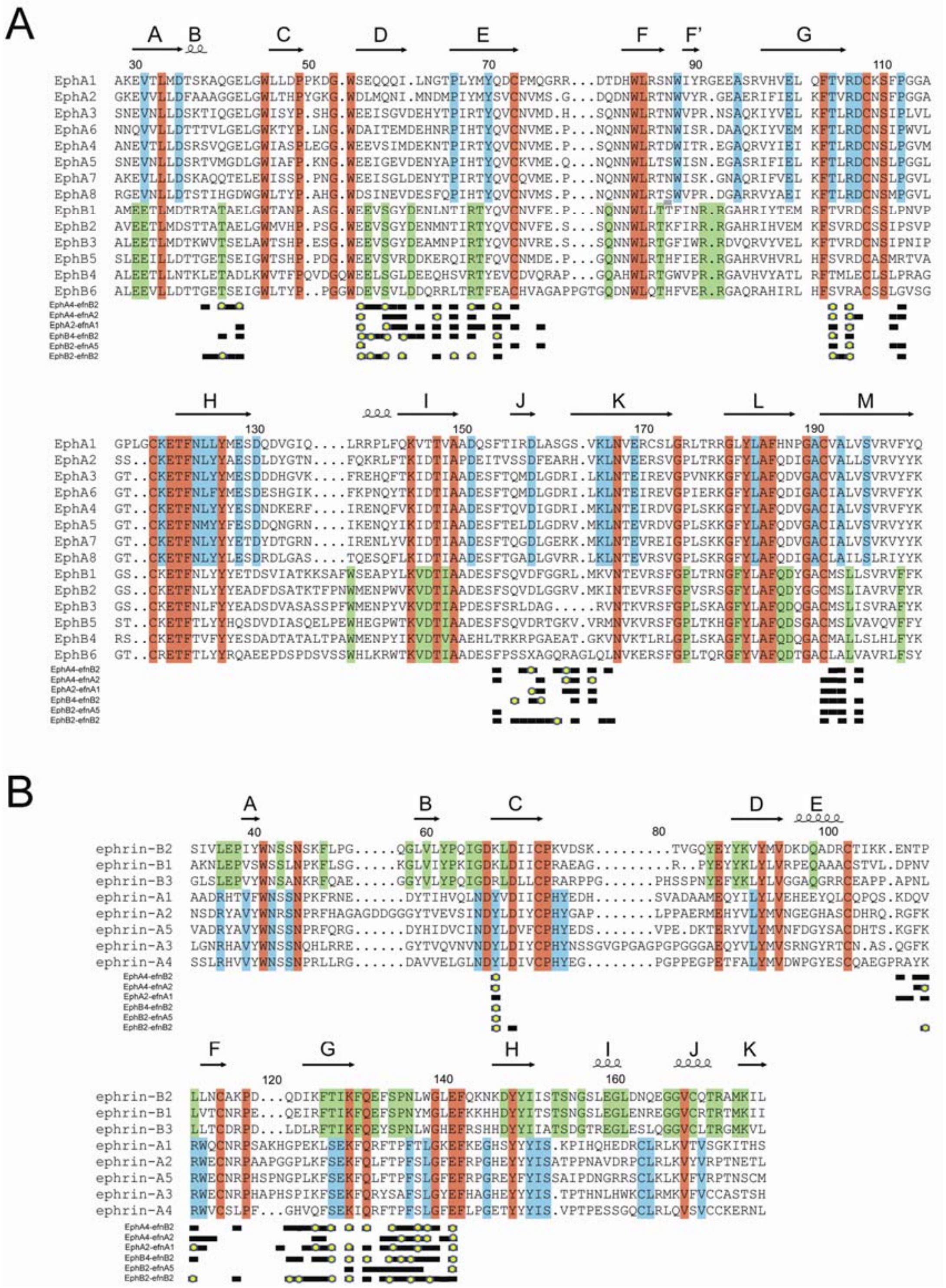

Figure S3. Structure-based Sequence Alignment of Human Eph Receptor LBD and Ephrin Ligand RBD

(A and B) Secondary structure elements are shown with an arrow (β-strand) and helix (α-helix) and are labelled according to standard Eph and ephrin nomenclature. Residues occluded in the receptor-ligand interfaces were determined by PDBsum (Laskowski, 2009) are highlighted by bars below the sequence. Residues participating in Eph–ephrin hydrogen bonds are highlighted by stars.

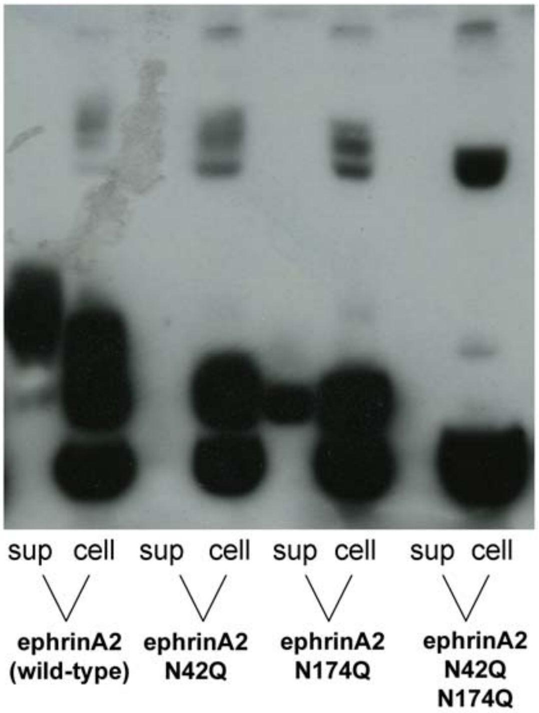

Figure S4. Test Expression of ephrinA2 Glycosylation Site Mutants in HEK 293T Cells by western blot
Protein was detected by mouse anti-His antibody (PentaHis, Qiagen) and IgG-Fc specific anti-mouse horseradish peroxidase (Sigma) according to standard methods. Lanes labelled “cell” and “sup” correspond to cell debris and cell supernatant, respectively. Single mutant N42Q^{ephrinA2} and double mutant N42Q^{ephrinA2}, N174Q^{ephrinA2} showed no secreted expression while single mutant N174Q^{ephrinA2} was secreted at similar levels to the wild-type ephrinA2 construct.

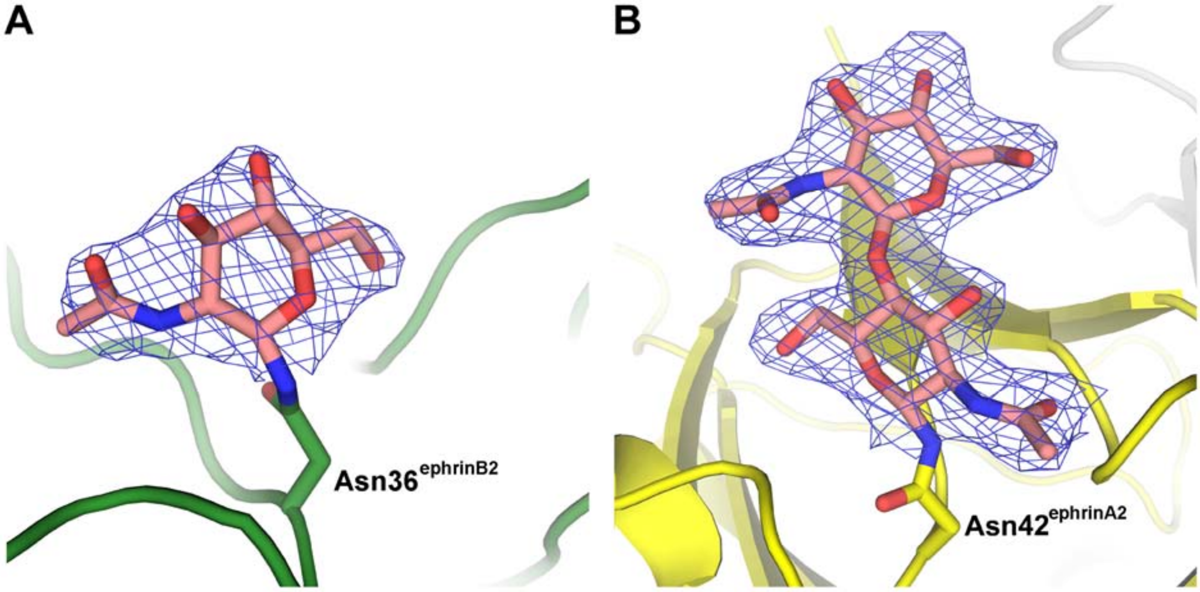

Figure S5. *N*-linked Glycosylation Sites on ephrinB2 and ephrinA2
(A) Cartoon representation of ephrinB2 with the GlcNAcβ1–Asn36 structure shown as sticks.
(B) Cartoon representation of ephrinA2 with the GlcNAcβ2,1–Asn42 structure shown as sticks. The carbons of GlcNAc are shown in pink, while those of the protein side chains are coloured green in panel A and yellow in panel B. Oxygen atoms are coloured red, and nitrogen atoms are blue. A 2F_o-F_c electron density map is displayed around the GlcNAc residues contoured at 1σ.

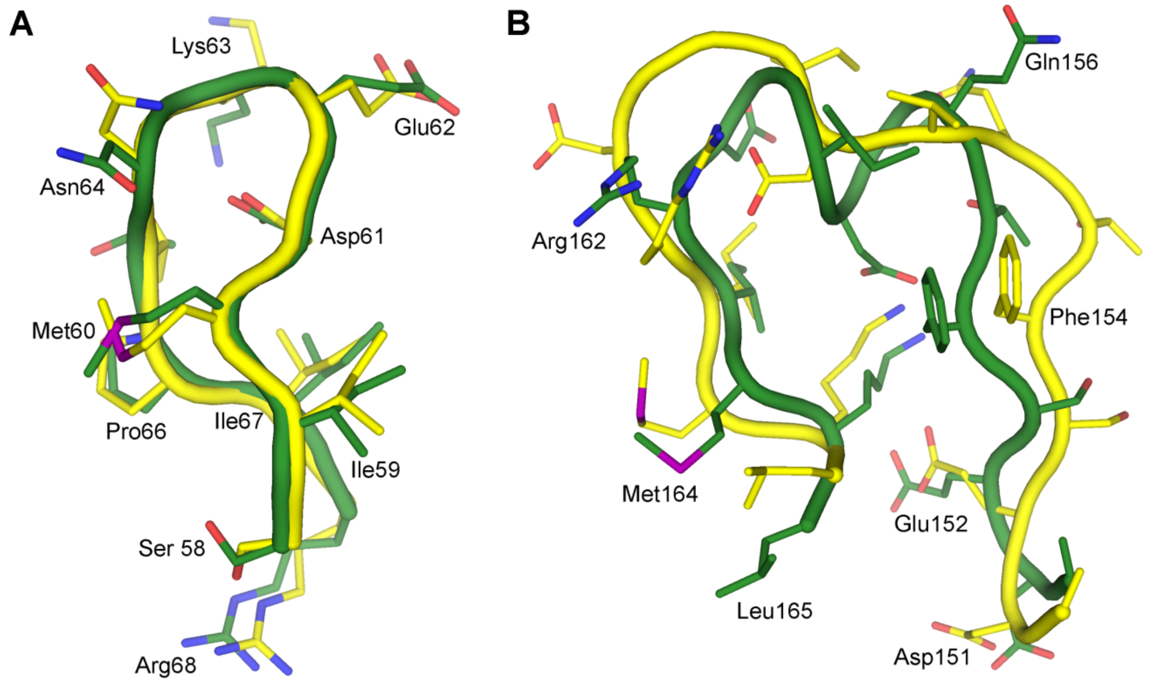

Figure S6. Primary Interaction Loops of the EphA4 Receptor
(A and B) The DE^{EphA4} (A) and JK^{EphA4} (B) loops from EphA4–ephrinB2 and EphA4–ephrinA2. The Cα mainchain of EphA4 from EphA4–ephrinB2 is shown in yellow and the Cα mainchain of EphA4 from EphA4–ephrinA2 is shown in green. Oxygen atoms are coloured red, sulphur atoms are coloured purple, and nitrogen atoms are blue.

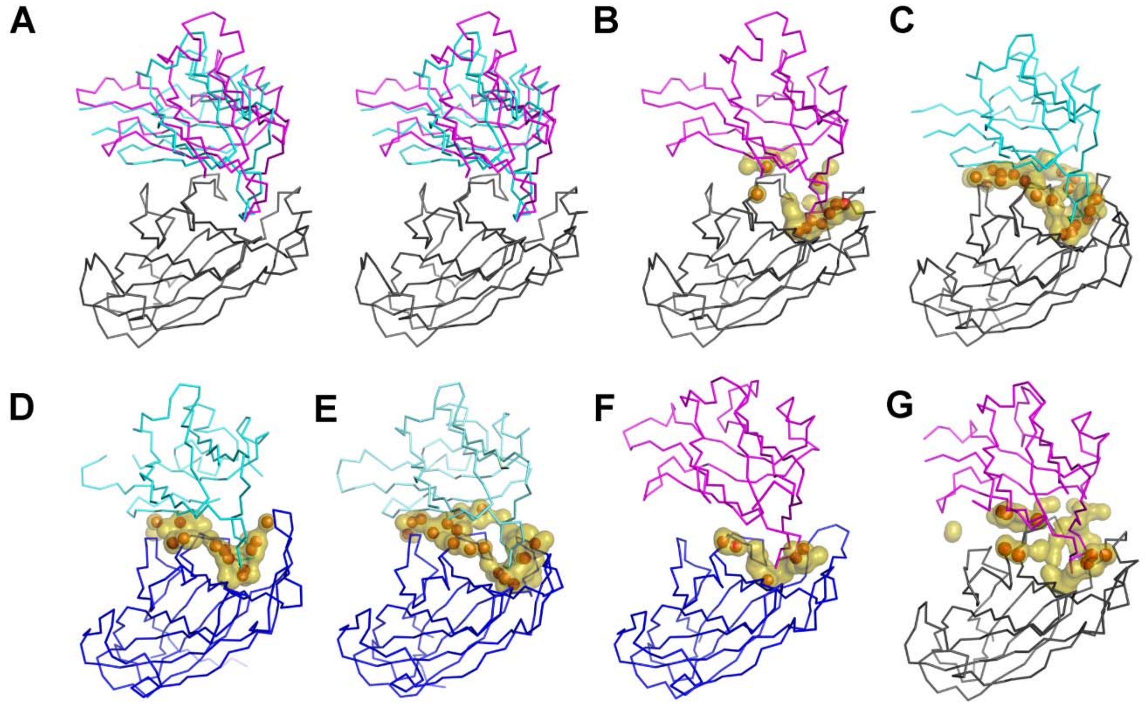

Figure S7. Ephrin-class Dependent Binding
(A) Comparison of ephrinA2 (pink) and ephrinB2 (cyan) bound to EphA4 (gray) based on superposition of the EphA4 component (shown in stereo representation) reveals a 12° relative tilt between ephrinA– and ephrinB–bound complexes.
(B–G) The tilt of ephrin binding to its receptor is dependent upon the degree of Van der Waals and hydrogen bonding interactions. ephrinB class ligands are coloured cyan and ephrinA class ligands are coloured pink. Mid-points between Van der Waals interactions are shown as gold surfaces and inter-complex hydrogen bonds are shown as red spheres.

Complexes are presented: (B) EphA4–ephrinA2, (C) EphA4–ephrinB2, (D) EphB4–ephrinB2 (PDB code 2HLE), (E) EphB2–ephrinB2 (PDB code 1KGY), (F) EphB2–ephrinA5 (PDB code 1SHW) and (G) EphA2–ephrinA1 (PDB code 3CZU).

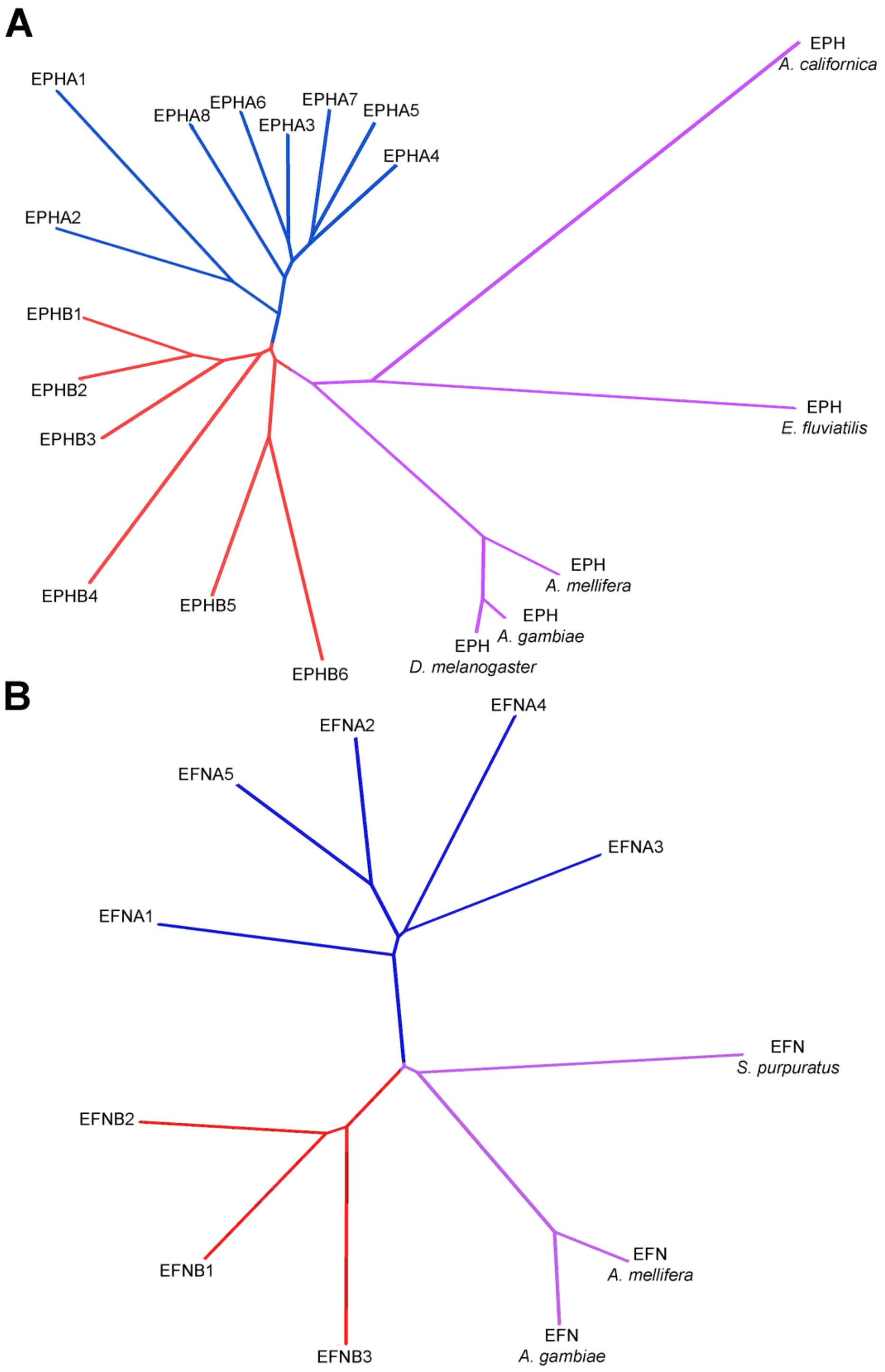

Figure S8. Sequence-based Phylogenetic Analysis of Eph Receptors and ephrin Ligands
(A) Unrooted phylogenetic tree of the LBD of Eph receptors.
(B) Unrooted phylogenetic tree of the RBD of ephrin ligands. Trees were generated using the programs ClustalW (Chenna et al., 2003), and Quicktree (Felsenstein, 1989). All sequences shown are human unless labelled otherwise. Branches coloured blue correspond to sequences of A-class Eph and ephrin proteins, branches coloured red correspond to sequences of B-class Eph and ephrin proteins, and branches coloured lilac correspond to a selection of Eph and ephrin proteins from lower order vertebrates and invertebrates.

Table S1. Crystallization Data for EphA4, EphA4–ephrinA2, and EphA4–ephrinB2

Structure	Conc. (mg/ml)	Condition	Temp. (Celsius)	Time (Day)	Solvent Content (%)	Cryo Protectant
EphA4	7	30% PEG 4000, 8% propan-1-ol, and 100mM Tris pH 8.5	21	5	48.8	PFO-X125/03 oil
EphA4–ephrinB2	12	30% PEG 6000, 1 M Lithium Chloride, and 100 mM MES pH 6.0	21	4	42.4	25% glycerol
EphA4–ephrinA2	29	20% PEG 3350, 200 mM KNO_3	4	3	59.3	30% PEG 400

SUPPLEMENTAL REFERENCES

Chenna, R., Sugawara, H., Koike, T., Lopez, R., Gibson, T.J., Higgins, D.G., and Thompson, J.D. (2003). Multiple sequence alignment with the Clustal series of programs. Nucleic Acids Res *31*, 3497-3500.

Felsenstein, J. (1989). PHYLIP -- Phylogeny Inference Package (version 3.2). Cladistics *5*, 164-166.

Laskowski, R.A. (2009). PDBsum new things. Nucleic Acids Res *37*, D355-359.
